# Supplementary material for: Distinct mechanisms regulate ventricular and atrial chamber wall formation
Source: Nat Commun. 2024 Sep 17;15:8159. doi: 10.1038/s41467-024-52340-3 (PMC11408654; doi:10.1038/s41467-024-52340-3)
Supplement: Supplementary file 1 — Supplementary Information [file 41467_2024_52340_MOESM1_ESM.pdf]

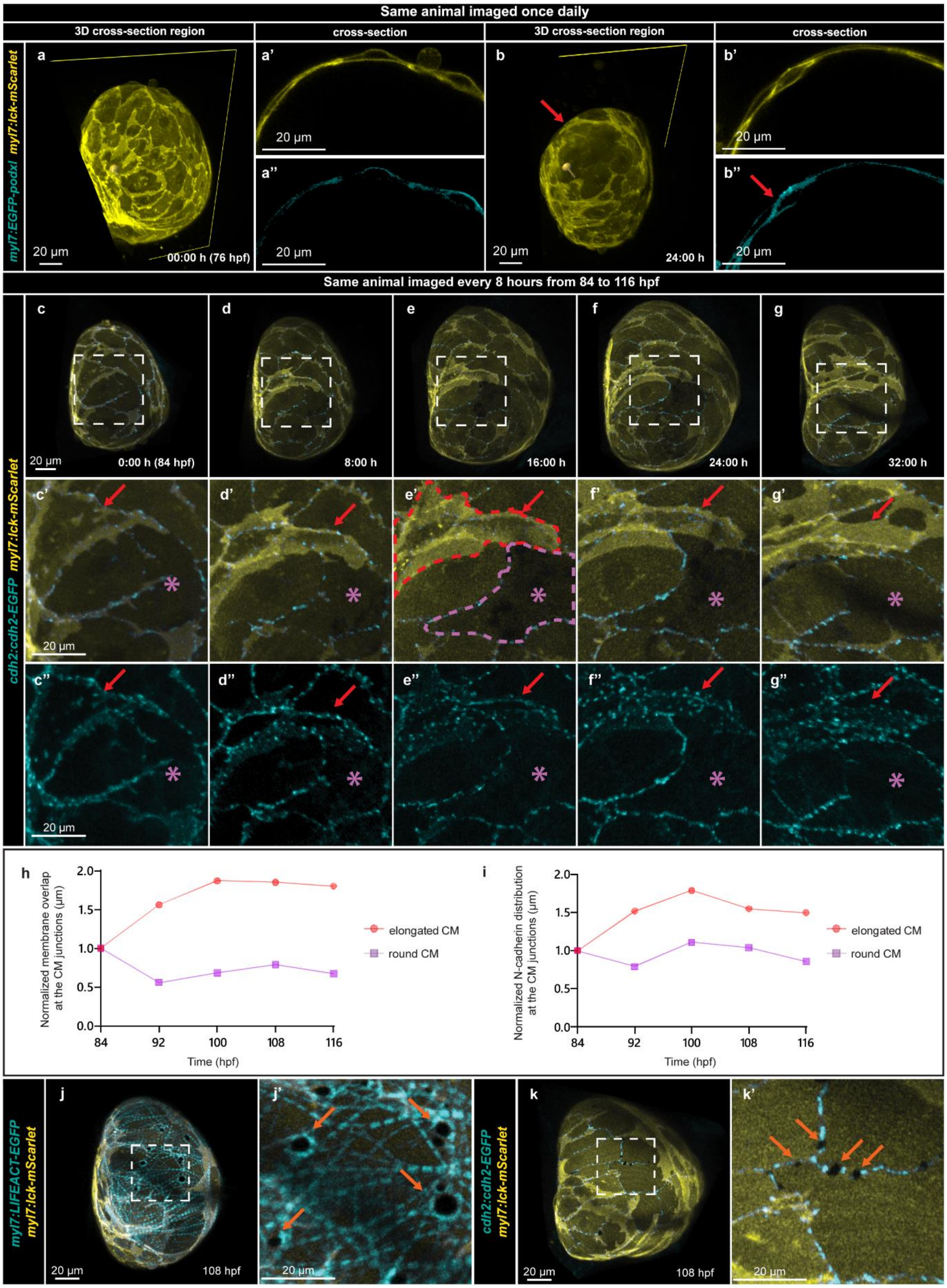

Same animal imaged once daily
3D cross-section region
cross-section
3D cross-section region
cross-section
myl7:EGFP-podxl myl7:lck-mScarlet
a
a'
a''
b
b'
b''
20 µm
00:00 h (76 hpf)
24:00 h
Same animal imaged every 8 hours from 84 to 116 hpf
cdh2:cdh2-EGFP myl7:lck-mScarlet
c
d
e
f
g
0:00 h (84 hpf)
8:00 h
16:00 h
24:00 h
32:00 h
c'
d'
e'
f'
g'
c''
d''
e''
f''
g''
h
Normalized membrane overlap at the CM junctions (µm)
Time (hpf)
elongated CM
round CM
i
Normalized N-cadherin distribution at the CM junctions (µm)
Time (hpf)
elongated CM
round CM
myl7:LIFEACT-EGFP myl7:lck-mScarlet
j
j'
108 hpf
cdh2:cdh2-EGFP myl7:lck-mScarlet
k
k'
108 hpf

Supplementary Figure 1: Atrial cardiomyocyte intercalation changes apicobasal polarity and N-cadherin distribution. a-b'', 3D cross-section regions of confocal images taken from the same atrium at 76 and 100 hpf; CM membranes shown in yellow (*myl7:lck-mScarlet*) and Podocalyxin in cyan (*myl7:EGFP-podxl*); 2D cross-sections (**a', a'', b', b''**); red arrows point to elongating atrial CM. **c-g'',** Airyscan images of the same larva imaged every 8 hours from 84 to 116 hpf; CM membranes shown in yellow (*myl7:lck-mScarlet*) and N-cadherin in cyan (*cdh2:cdh2-EGFP*); white squares indicate the zoomed in regions; red arrows point to an elongating CM; purple asterisk marks a round CM; red and purple dashed lines in c' outline the analyzed CMs. **h, i,** Changes in the distribution of the mScarlet membrane marker and of N-cadherin in the elongating (red) and round (purple) CMs. **j, j',** Airyscan images of a 108 hpf atrium with CM membranes in yellow (*myl7:lck-mScarlet*) and CM actin in cyan (*myl7:LIFEACT-EGFP*); white square indicates zoomed in region; arrows point to CM membrane detachments. **k, k',** Airyscan images of a 108 hpf atrium with CM membranes in yellow (*myl7:lck-mScarlet*) and N-cadherin in cyan (*cdh2:cdh2-EGFP*); white square indicates zoomed in region; arrows point to CM membrane detachments.

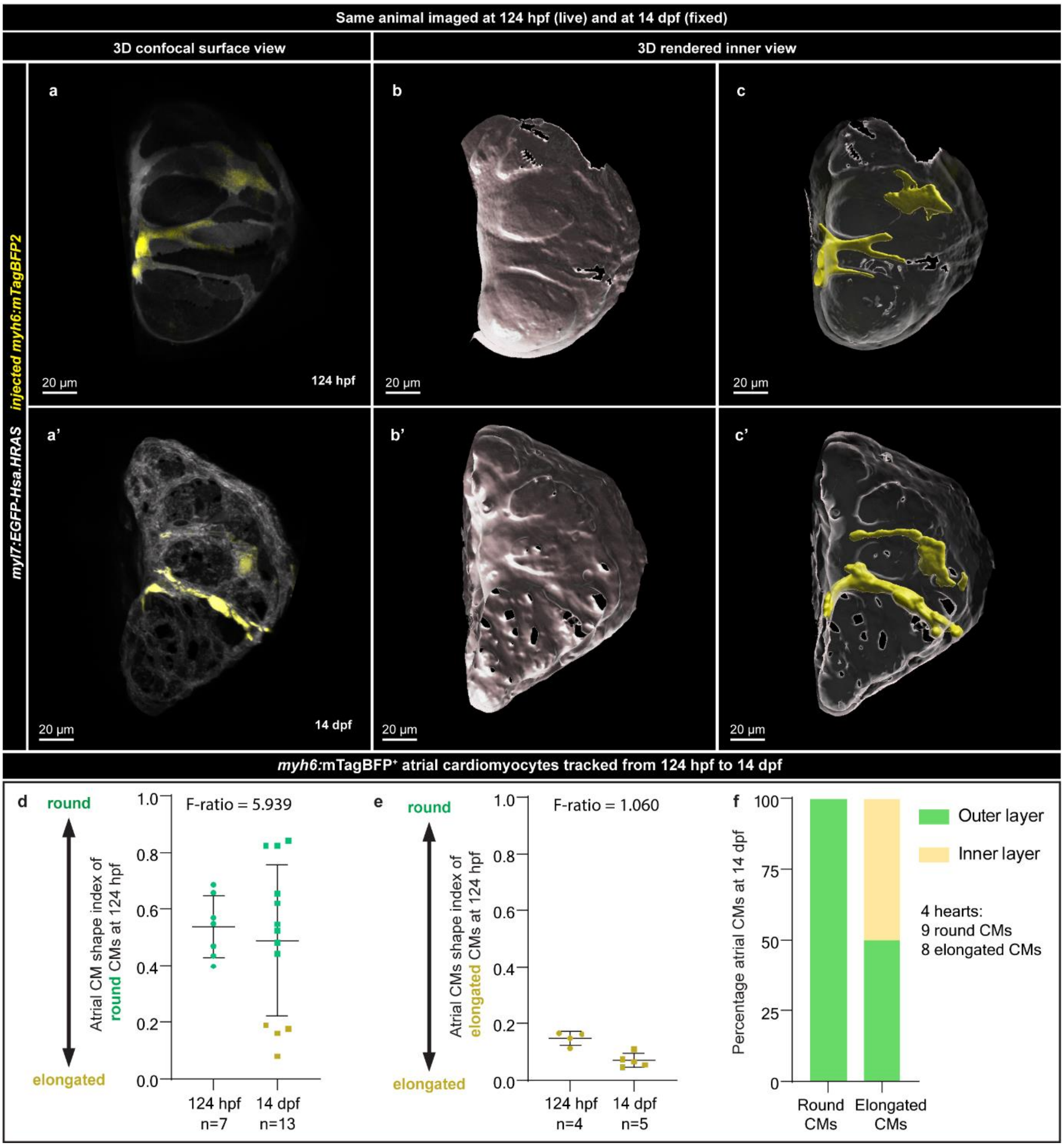

Same animal imaged at 124 hpf (live) and at 14 dpf (fixed)
3D confocal surface view
3D rendered inner view
myl7:EGFP-Hsa.HRAS
injected myh6:mTagBFP2
a
b
c
a'
b'
c'
20 µm
124 hpf
14 dpf
myh6:mTagBFP+ atrial cardiomyocytes tracked from 124 hpf to 14 dpf
d
round
elongated
Atrial CM shape index of round CMs at 124 hpf
F-ratio = 5.939
1.0
0.8
0.6
0.4
0.2
0.0
124 hpf n=7
14 dpf n=13
e
round
elongated
Atrial CMs shape index of elongated CMs at 124 hpf
F-ratio = 1.060
124 hpf n=4
14 dpf n=5
f
Percentage atrial CMs at 14 dpf
100
75
50
25
0
Outer layer
Inner layer
4 hearts:
9 round CMs
8 elongated CMs
Round CMs
Elongated CMs

Supplementary Figure 2: Only a subset of atrial cardiomyocytes form the inner muscle structures. a, a’, 3D airyscan imaging of the same atrium at 124 hpf (live) and 14 dpf (fixed); CM membranes shown in white (*myl7:EGFP-Hsa.HRAS*) and mosaic CM cytoplasmic marker in yellow (*-0.8myl7:mTagBFP2*). **b, b’,** 3D surface rendering of the membrane marker from a, a’. **c, c’,** 3D surface rendering of both the membrane (transparent surface) and cytoplasmic (opaque surface) signals from the images shown in a and a’. **d, e**, Shape index of *myh6:*mTagBFP^{+} CMs from experiment a-c’, determined from the same animals imaged at 124 hpf and 14 dpf (n=7 round CMs at 124 hpf and n=13 CMs at 14 dpf from the 7 round CMs mTagBFP^{+} at 124 hpf and their daughter cells; n=4 elongating CMs at 124 hpf and n=5 elongated CMs at 14 dpf from the 7 elongated CMs mTagBFP^{+} at 124 hpf and their daughter cells; F-test from two-tailed unpaired Student’s t-test, p=0.0389 (d), p=0.1485 (e)). **f,** Percentage of round or elongating atrial CMs localized in the outer or inner layer of 14 dpf atrium (n=4 hearts, 17 mTagBFP^{+} CMs were quantified, 9 round, 8 elongating). Error bars are mean ± SD.

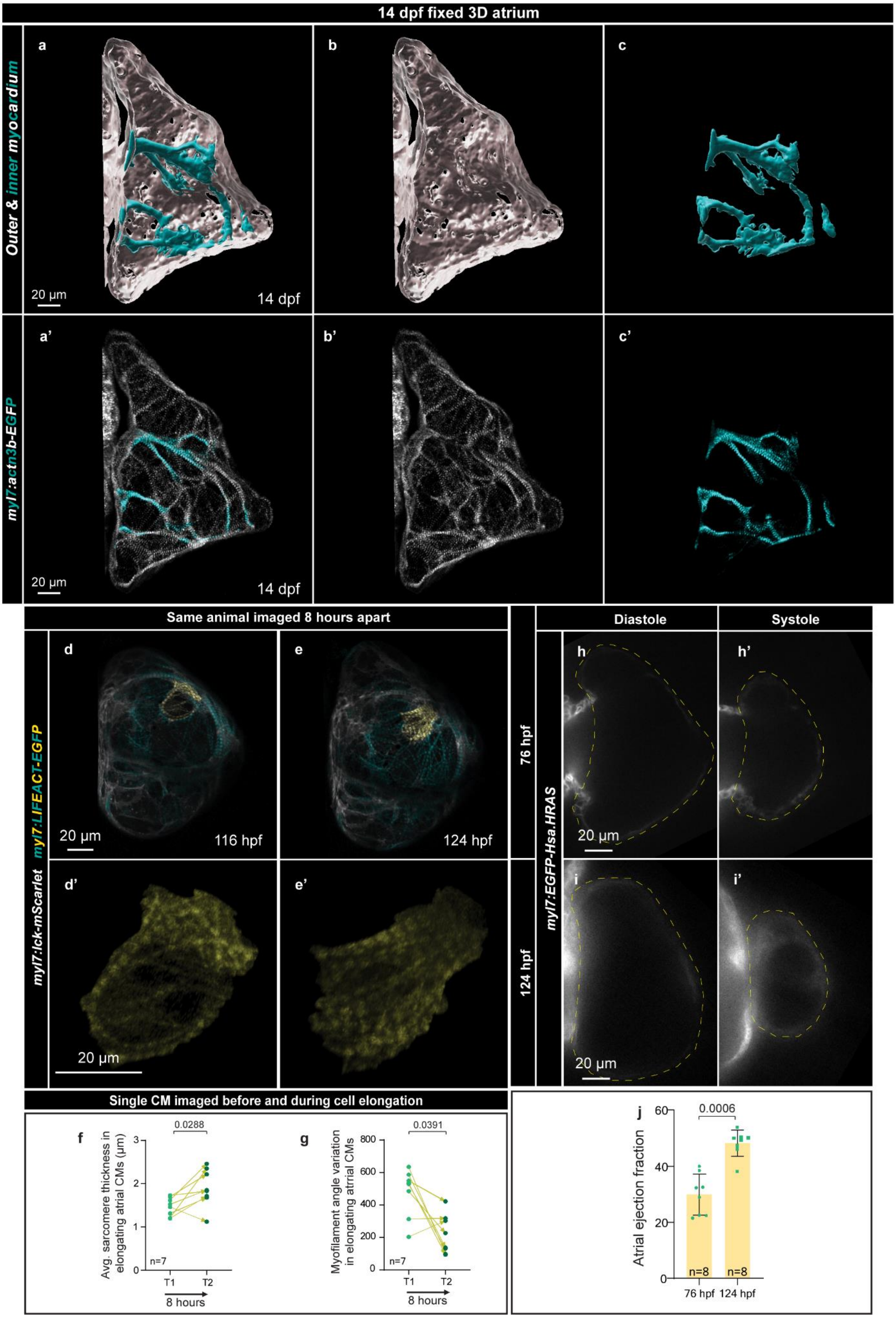
14 dpf fixed 3D atrium
Outer & inner myocardium
myl7:actn3b-EGFP
a
b
c
a'
b'
c'
20 µm
14 dpf
Same animal imaged 8 hours apart
myl7:lck-mScarlet myl7:LIFEACT-EGFP
d
e
d'
e'
116 hpf
124 hpf
Diastole
Systole
76 hpf
124 hpf
myl7:EGFP-Hsa.HRAS
h
h'
i
i'
Single CM imaged before and during cell elongation
f
Avg. sarcomere thickness in elongating atrial CMs (µm)
0.0288
n=7
T1
T2
8 hours
g
Myofilament angle variation in elongating atrial CMs
0.0391
j
Atrial ejection fraction
0.0006
n=8
76 hpf
124 hpf

Supplementary Figure 3: Correlation between atrial cardiomyocyte elongation and myofibril maturation. **a-c,** 3D surface rendering of a fixed atrium at 14 dpf; outer layer myocardium shown in white and inner layer myocardium in cyan. **a'-c',** 3D confocal images of the atrium from which the surface rendering was created; sarcomere Z-bands shown in white for outer layer CMs and in cyan for inner layer CMs (*myl7:actn3b-EGFP*). **d-e',** 3D confocal images of the same atrium before (116 hpf) and during (124 hpf) CM elongation; CM membranes shown in grey (*myl7:lck-mScarlet*) and CM actin in cyan (*myl7:LIFEACT-EGFP*). Example of myofibrils from an elongating CM tracked over time (yellow). **f, g,** Graphs showing the changes in sarcomere thickness and myofilament angle over time in elongating CMs (n=7; each data point represents the average value in one elongating CM; two tailed paired Student's t-test (f), two-tailed Wilcoxon test (g)). **h-i',** Images from 2D spinning disk microscopy of a beating atrium at 76 (h, h') and 124 (i, i') hfp (different animals shown); CM membranes shown in grey (*myl7:EGFP-Hsa.HRAS*). **j,** Graph of atrial ejection fraction measured at 76 (prior to CM elongation) and 124 (after CM elongation) hpf (n=8 at 76 hpf and n=8 at 124 hpf; each data point represents one heart; two-tailed Mann-Whitney test) (different zebrafish used). Error bars are mean ± SD.

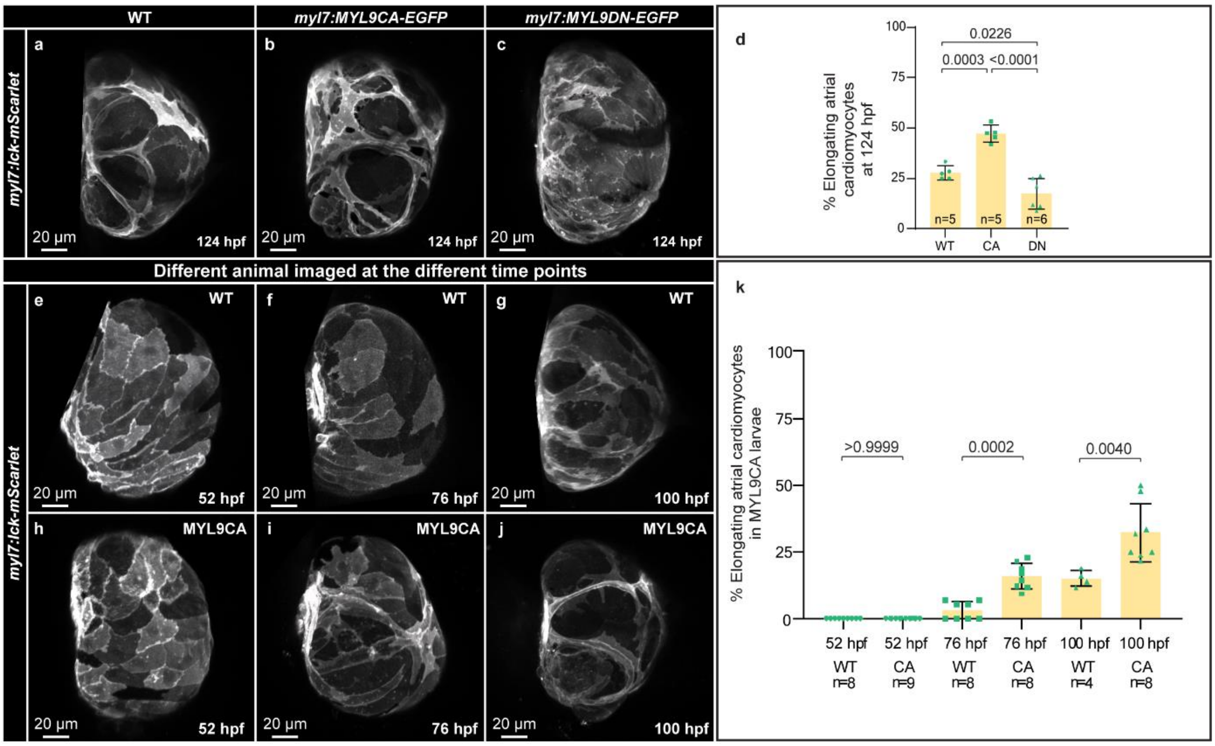

Extended Data Fig. 4: The extent of atrial cardiomyocyte elongation is regulated by cell contractility. **a-c,** 3D airyscan images of atria from 124 hpf wild-type, MYL9^{CA} overexpressing (*myl7:MYL9CA-EGFP*), and MYL9^{DN} overexpressing (*myl7:MYL9DN-EGFP*) larvae. CM membranes shown in white (*myl7:lck-mScarlet*). **d,** Percentage of elongating atrial CMs at 124 hpf in wild-type, MYL9^{CA} and MYL9^{DN} overexpressing hearts (n=5 wild-type and CA and n=6 DN; each data point represents one heart; ordinary one-way ANOVA with Tukey's multiple comparisons test, CA vs DN p-value = $0.23*10^{-5}$). **e-g,** 3D airyscan images of wild-type and MYL9^{CA} overexpressing hearts (*myl7:MYL9CA-EGFP*) atria. The images are from different larvae taken at 52, 76 and 100 hpf. CM membranes shown in white (*myl7:lck-mScarlet*). **k,** Percentage of elongating atrial CMs in 52, 76 and 100 hpf wild-type and MYL9^{CA} hearts (n=8 52 hpf wild-type, n=9 52 hpf CA, n=8 76 hpf wild-type, n=8 76 hpf CA, n=4 100 hpf wild-type, n=8 100 hpf CA; each data point represents one heart; two-tailed Mann-Whitney test for each two conditions compared). Error bars are mean ± SD.

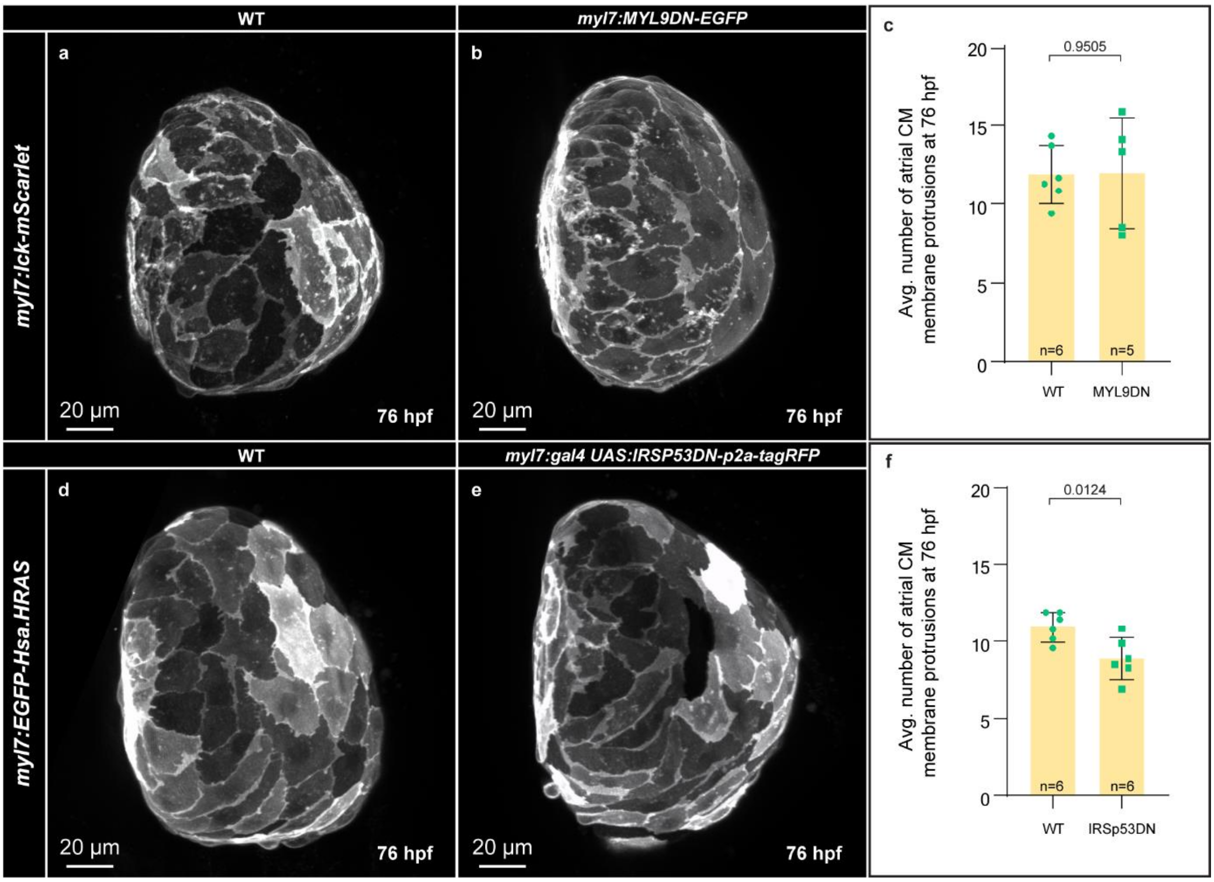

Supplementary Figure 5: Cellular contractility does not appear to affect the number of membrane protrusions in atrial cardiomyocytes. a, b, 3D airyscan images of the atrium from 76 hpf wild-type (a) and CM specific MYL9^{DN} overexpressing (b) siblings; CM membranes shown in white (*myl7:lck-mScarlet*). **c,** Number of membrane protrusions per atrial CM and averaged per heart in 76 hpf from wild-type and CM specific MYL9^{DN} overexpressing siblings (n=6 wild-type hearts; n=5 MYL9^{DN} overexpressing hearts; each value represents the average from one heart; two-tailed unpaired Student's t-test). **d, e,** 3D airyscan images of 76 hpf atrium from wild-type (a) and CM specific IRSP53^{DN} overexpressing (b) siblings; CM membranes shown in white (*myl7:EGFP-Hsa.HRAS*). **f,** Number of membrane protrusions per cell and averaged per atrial CM and averaged per heart in 76 hpf from wild-type and CM specific IRSp53^{DN} overexpressing siblings (n=6 wild-type hearts; n=6 IRSp53^{DN} overexpressing hearts; each value represents the average from one heart; two-tailed unpaired Student's t-test). Error bars are mean ± SD.

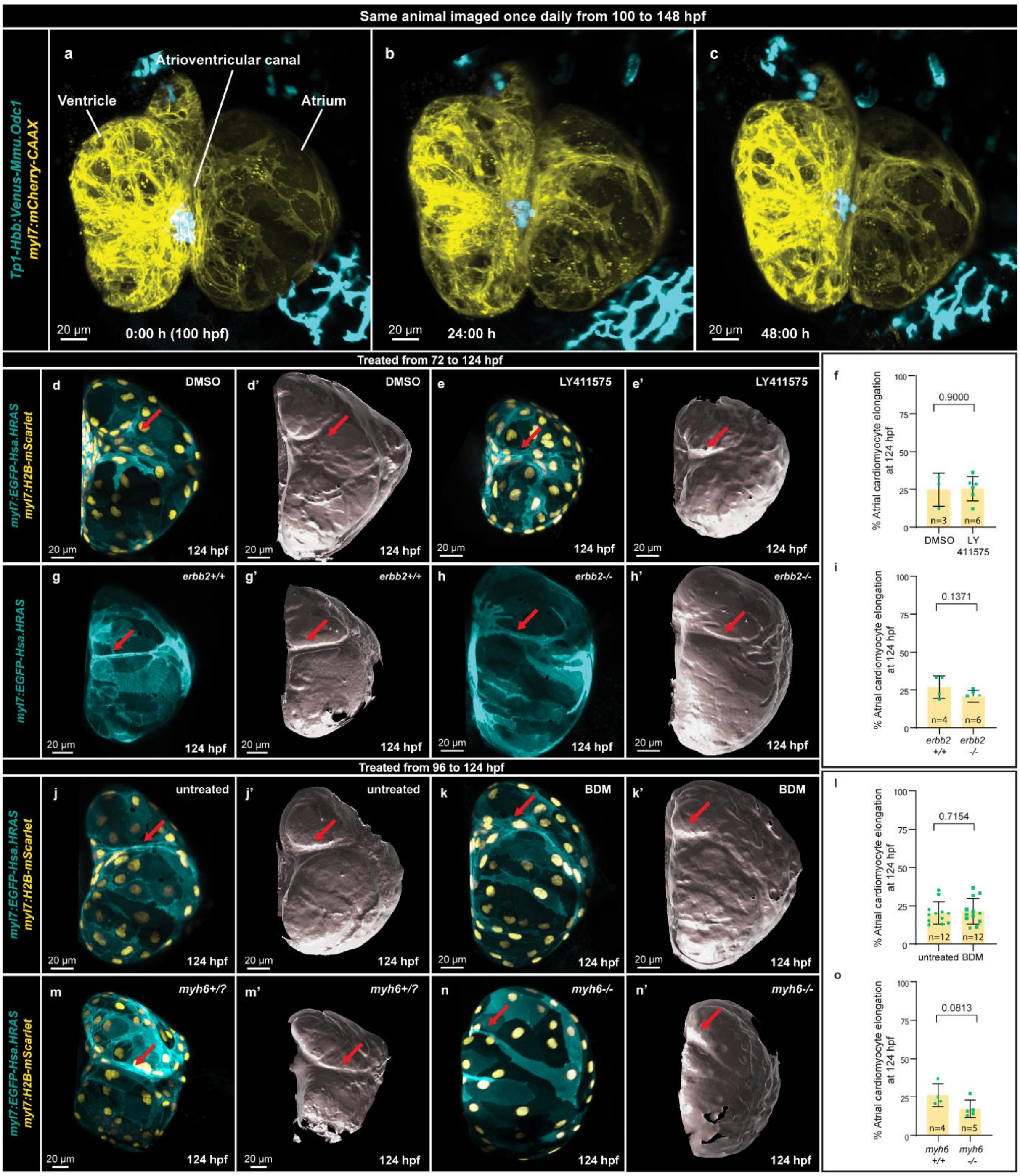

Same animal imaged once daily from 100 to 148 hpf
Tp1-Hbb:Venus-Mmu.Odc1
myl7:mCherry-CAAX
a
Atrioventricular canal
Ventricle
Atrium
20 µm
0:00 h (100 hpf)
b
24:00 h
c
48:00 h
Treated from 72 to 124 hpf
myl7:EGFP-Hsa.HRAS
myl7:H2B-mScarlet
d
DMSO
d'
e
LY411575
e'
124 hpf
f
% Atrial cardiomyocyte elongation at 124 hpf
0.9000
n=3
n=6
DMSO
LY 411575
g
erbb2+/+
g'
h
erbb2-/-
h'
i
0.1371
n=4
erbb2 +/+
erbb2 -/-
Treated from 96 to 124 hpf
j
untreated
j'
k
BDM
k'
l
0.7154
n=12
untreated BDM
m
myh6+/?
m'
n
myh6-/-
n'
o
0.0813
n=5
myh6 +/+
myh6 -/-

Supplementary Figure 6: Processes required for ventricular trabeculation are not required for atrial morphogenesis. a-c, 3D longitudinal confocal images of 100, 124, and 148 hpf hearts; CM membranes shown in yellow (*myl7:mCherry-CAAX*) and Notch reporter expression in cyan (*Tp1-Hbb:Venus-Mmu.Odc1*). **d, e,** 3D airyscan imaging of atria from 124 hpf larvae treated from 72 to 124 hpf with DMSO or LY411575; red arrows point to elongating CMs. **d', e'** Cross-sections through 3D myocardial surface renderings of (d) and (e); arrows point to inner ridges corresponding to the elongating CMs from (d) and (e). **f,** Percentage of elongating atrial CMs from 124 hpf larvae treated with DMSO or LY411575 from 72 to 124 hpf (n=3 DMSO; n=6 LY411575; each data point represents one heart; two-tailed unpaired Student's t-test). **g, h,** 3D airyscan images of atria from 124 hpf homozygous wild-type and *erbb2* mutant sibling larvae; CM membranes shown in cyan (*myl7:EGFP-Hsa.HRAS*); red arrows point to elongating CMs. **g', h'** Cross-sections through 3D myocardial surface renderings of (g) and (h); arrows point to inner ridges corresponding to the elongating CMs from (g) and (h). **i,** Percentage of elongating atrial CMs from 124 hpf homozygous wild-type and *erbb2* mutant siblings (n=4 wild-type; n=6 *erbb2* mutants, each data point represents one heart; two-tailed unpaired Student's t-test). **j, k,** 3D airyscan imaging of atria from 124 hpf larvae treated from 96 to 124 hpf with DMSO or BDM; CM membranes shown in cyan (*myl7:Hsa.HRAS-EGFP*) and CM nuclei in yellow (*myl7:H2B-mScarlet*); red arrows point to elongating CMs. **j', k'** Cross-sections through 3D myocardial surface renderings of (j) and (k); arrows point to inner ridges corresponding to the elongating CMs from (j) and (k). **l,** Percentage of elongating atrial CMs from 124 hpf untreated and BDM treated (n=12 untreated; n=12 BDM; each data point represents one heart; two-tailed unpaired Student's t-test). **m, n,** 3D airyscan imaging of 124 hpf homozygous wild-type and *myh6* mutant siblings; CM membranes shown in cyan (*myl7:EGFP-Hsa.HRAS*) and CM nuclei in yellow (*myl7:H2B-mScarlet*); red arrows point to elongating CMs. **m', n'** Cross-sections through 3D myocardial surface renderings of (m) and (n); arrows point to inner ridges corresponding to elongating CMs from (m) and (n). **o,** Percentage of elongating atrial CMs from 124 hpf homozygous wild-type and *myh6* mutant sibling larvae (n=4 wild-type; n=5 *myh6* mutants; each data point represents one heart; two-tailed unpaired Student's t-test). Error bars are mean ± SD.

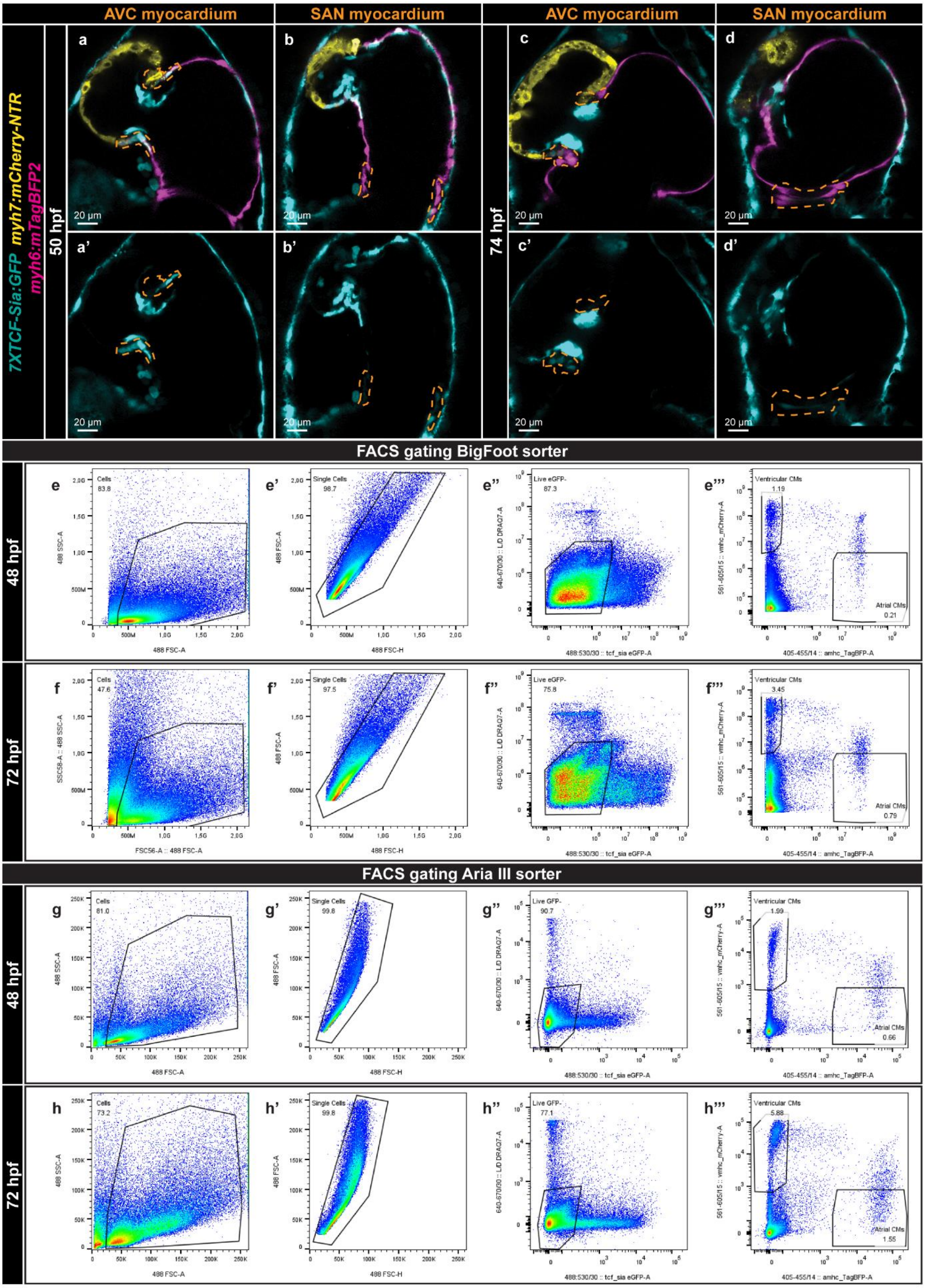
AVC myocardium
SAN myocardium
AVC myocardium
SAN myocardium
7XTCF-Sia:GFP myh7:mCherry-NTR myh6:mTagBFP2
50 hpf
74 hpf
20 µm
FACS gating BigFoot sorter
48 hpf
72 hpf
Cells
Single Cells
Live eGFP-
Ventricular CMs
Atrial CMs
488 FSC-A
488 SSC-A
488 FSC-H
488:530/30 :: tcf_sia eGFP-A
640-670/30 :: L/D DRAQ7-A
561-605/15 :: vmhc_mCherry-A
405-455/14 :: amhc_TagBFP-A
FACS gating Aria III sorter
Live GFP-

Extended Data Fig. 7: Atrial cardiomyocyte Fluorescence Activated Cell Sorting. a-d', Confocal images of a zebrafish heart at 50 (a-b') and 74 (c-d') hpf; ventricular CMs shown in yellow (*myh7:mCherry-NTR*), atrial CMs in magenta (*-4.5myh6:mTagBFP2*), and AVC and sinoatrial node CMs in cyan (*7XTCF-Sia:eGFP*); AVC and sinoatrial node CMs outlined by the orange dashed lines (a'-d'). **e-f'''**, Graphs showing the BigFoot gating strategies to sort atrial CMs at 48 (e-e''') and 72 (f-f''') hpf. **g-h'''**, Graphs showing the Aria III gating strategies to sort atrial CMs at 48 (g-g''') and 72 (h-h''') hpf.

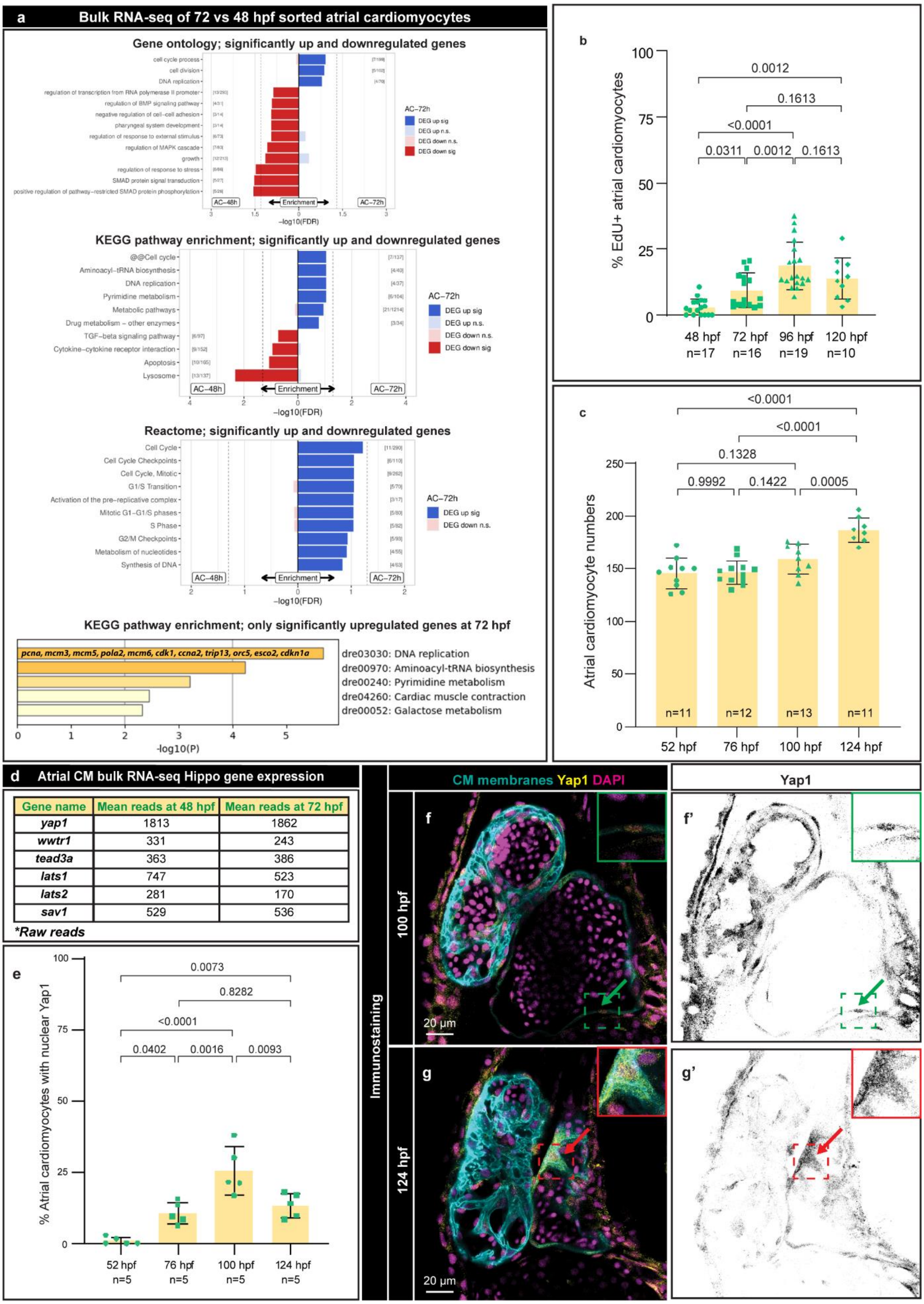
a
Bulk RNA-seq of 72 vs 48 hpf sorted atrial cardiomyocytes
Gene ontology; significantly up and downregulated genes
cell cycle process
cell division
DNA replication
regulation of transcription from RNA polymerase II promoter
regulation of BMP signaling pathway
negative regulation of cell-cell adhesion
pharyngeal system development
regulation of response to external stimulus
regulation of MAPK cascade
growth
regulation of response to stress
SMAD protein signal transduction
positive regulation of pathway-restricted SMAD protein phosphorylation
AC-72h
DEG up sig
DEG up n.s.
DEG down n.s.
DEG down sig
AC-48h
Enrichment
-log10(FDR)
KEGG pathway enrichment; significantly up and downregulated genes
@@Cell cycle
Aminoacyl-tRNA biosynthesis
DNA replication
Pyrimidine metabolism
Metabolic pathways
Drug metabolism – other enzymes
TGF-beta signaling pathway
Cytokine-cytokine receptor interaction
Apoptosis
Lysosome
Reactome; significantly up and downregulated genes
Cell Cycle
Cell Cycle Checkpoints
Cell Cycle, Mitotic
G1/S Transition
Activation of the pre-replicative complex
Mitotic G1-G1/S phases
S Phase
G2/M Checkpoints
Metabolism of nucleotides
Synthesis of DNA
KEGG pathway enrichment; only significantly upregulated genes at 72 hpf
pcna, mcm3, mcm5, pola2, mcm6, cdk1, ccna2, trip13, orc5, esco2, cdkn1a
dre03030: DNA replication
dre00970: Aminoacyl-tRNA biosynthesis
dre00240: Pyrimidine metabolism
dre04260: Cardiac muscle contraction
dre00052: Galactose metabolism
-log10(P)
b
% EdU+ atrial cardiomyocytes
0.0012
0.1613
<0.0001
0.0311
0.0012
0.1613
48 hpf n=17
72 hpf n=16
96 hpf n=19
120 hpf n=10
c
Atrial cardiomyocyte numbers
<0.0001
<0.0001
0.1328
0.9992
0.1422
0.0005
n=11
n=12
n=13
n=11
52 hpf
76 hpf
100 hpf
124 hpf
d Atrial CM bulk RNA-seq Hippo gene expression
Gene name | Mean reads at 48 hpf | Mean reads at 72 hpf
yap1 | 1813 | 1862
wwtr1 | 331 | 243
tead3a | 363 | 386
lats1 | 747 | 523
lats2 | 281 | 170
sav1 | 529 | 536
*Raw reads
e
% Atrial cardiomyocytes with nuclear Yap1
0.0073
0.8282
<0.0001
0.0402
0.0016
0.0093
52 hpf n=5
76 hpf n=5
100 hpf n=5
124 hpf n=5
Immunostaining
CM membranes Yap1 DAPI
Yap1
100 hpf
124 hpf
f
f'
g
g'
20 µm
20 µm

Extended Data Fig. 8: Atrial cardiomyocyte cell division and Hippo pathway gene expression. **a,** Significantly differentially expressed genes in sorted atrial CMs at 72 hpf compared with 48 hpf. **b,** Percentage of EdU^+ atrial CMs after a 24-hour pulse starting at the indicated times (n=17 48 hpf, n=16 72 hpf, n=19 96 hpf, n=10 120 hpf; each data point represents one heart; ordinary one-way ANOVA with Tukey's multiple comparisons test). **c,** Atrial CM numbers at 52, 76, 100, and 124 hpf (n=11 52 hpf, n=12 76 hpf, n=13 100 hpf, n=11 124 hpf; each data point represents one heart; Kruskal-Walllis test with Dunn's multiple comparisons test). **d,** Hippo pathway gene expression levels from bulk RNA-sequencing data of sorted atrial CMs at 48 and 72 hpf. **e**, Percentage of atrial CMs with nuclear Yap1 at 52, 76, 100, and 124 hpf (n=5 all time points; each data point represents one heart; ordinary one-way ANOVA with Tukey's multiple comparisons test). **f-g',** Confocal images of hearts immunostained for CM membranes (anti-GFP, cyan) and Yap1 (anti-Yap1, yellow f, g; grey f', g'), and counterstained with DAPI (DNA, magenta) imaged at 100 and 124 hpf; green arrow points to an atrial CM with nuclear Yap1 localization; red arrow points to an elongating atrial CM that does not exhibit nuclear Yap1 localization; squares show the magnified regions. Error bars are mean ± SD.

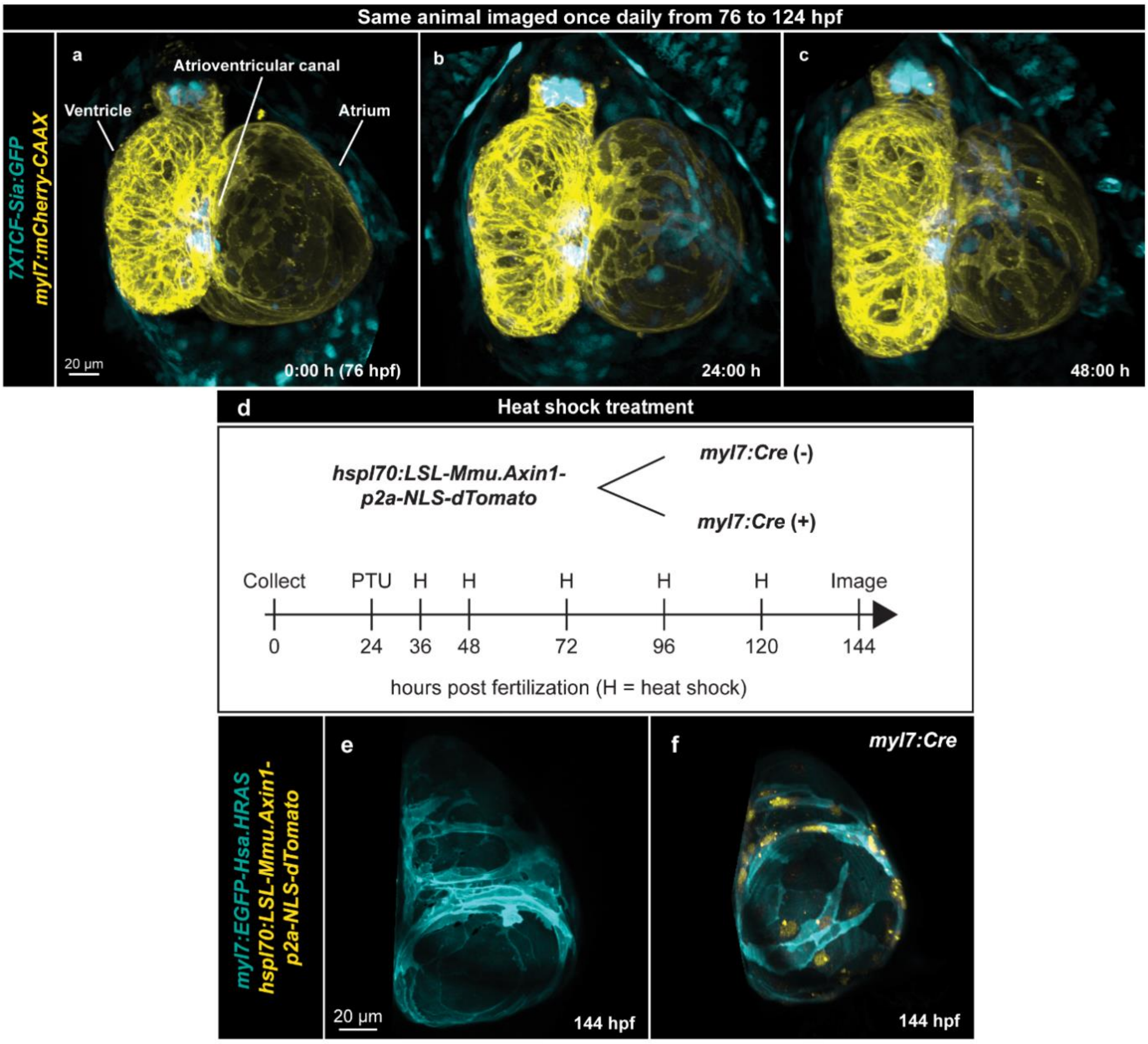

Supplementary Figure 9: No TCF-Sia reporter expression in atrial cardiomyocytes, and no significant effect on atrial cardiomyocyte elongation from overexpressing Axin1 in cardiomyocytes. **a-c,** 3D confocal imaging of the same heart taken once daily from 76 to 124 hpf; CM membranes shown in yellow (*myl7:mCherry-CAAX*) and Wnt/β-catenin reporter expression in cyan (*7xTCF-Sia:GFP*). **d**, Schematic of heat shock treatment (made in Adobe Illustrator by MA). **e, f,** 3D confocal images of atria from larvae that were heat shocked as described in (d); CM membranes shown in cyan (*myl7:EGFP-Hsa.HRAS*) and Axin1 overexpression in yellow (*hspl7:LSL-Mmu.Axin1-p2a-NLS-dTomato*).

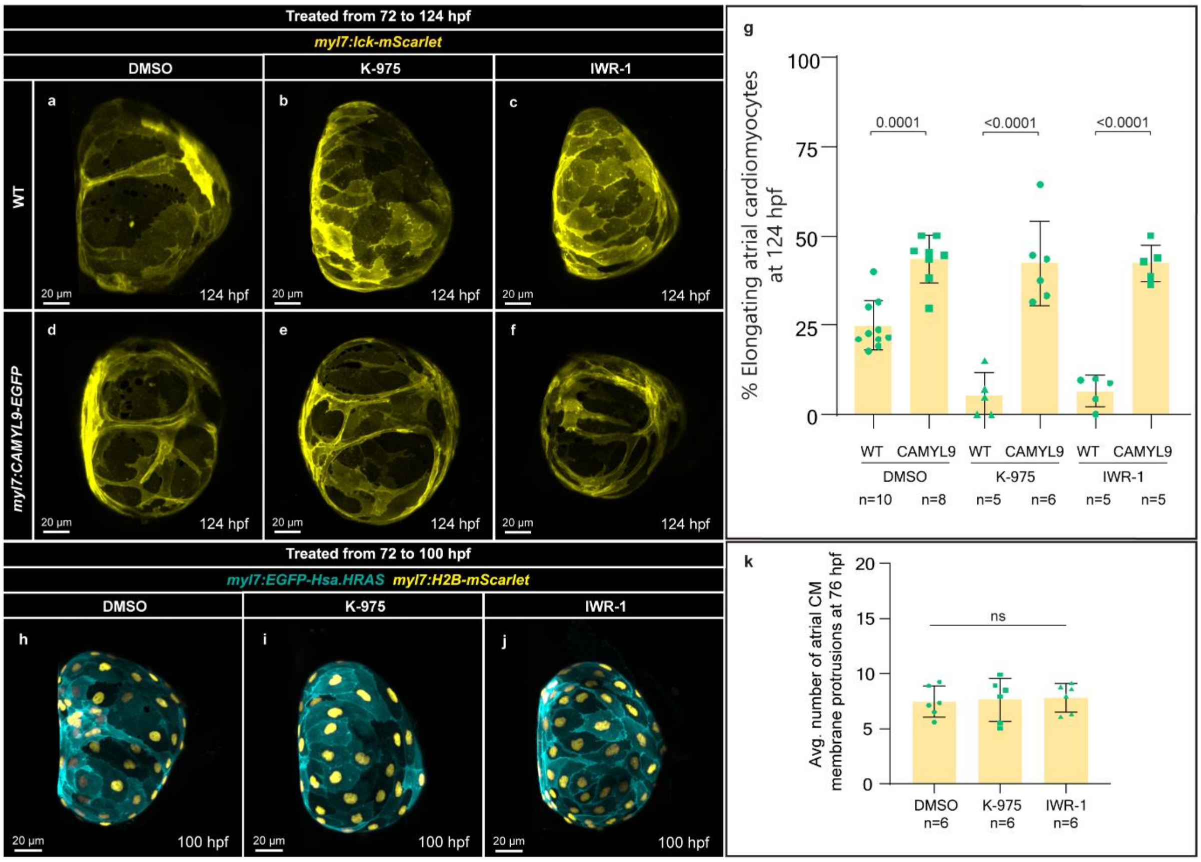

Supplementary Figure 10: Cytoskeletal changes important for atrial cardiomyocyte elongation occur downstream of the Hippo pathway. a-f, 3D confocal images of 124 hpf larvae treated from 72 to 124 hpf with DMSO, K-975, or IWR-1; CM membranes shown in yellow (*myl7:lck-mScarlet*); **a-c,** control siblings; **d-f**, CM specific MYL9^{CA} overexpressing larvae (*myl7: MYL9CA-EGFP*). **g,** Percentage of elongating atrial CMs from 124 hpf control siblings and CM specific MYL9^{CA} overexpressing larvae treated from 72 to 124 hpf with DMSO, K-975, or IWR-1 (n=10 DMSO control, n=8, DMSO MYL9^{CA}, n=5 K-975 control, n=6 K-975 MYL9^{CA}, n=5 IWR-1 control, n=5 IWR-1 MYL9^{CA}; each data point represents one heart; ordinary one-way ANOVA with Tukey's multiple comparisons test, K-975 WT vs MYL9^{CA} p-value = 0.28*10^{-8}, IWR-1 WT vs MYL9^{CA} p-value = 0.41*10^{-7}). **h-j,** 3D airyscan images of 100 hpf larvae treated from 72 to 100 hpf with DMSO, K-975, or IWR-1; CM membranes shown in cyan (*myl7:EGFP-Hsa.HRAS*) and CM nuclei in yellow (*myl7:H2B-mScarlet*). **k,** Number of atrial CM membrane protrusions determined per cell and averaged per heart (n=6 all three conditions; ordinary one-way ANOVA with Tukey's multiple comparisons test; DMSO vs K-975 p-value = 0.9780, DMSO vs IWR-1 p-value = 0.9113, K-975 vs IWR-1 p-value = 0.9758). Error bars are mean ± SD.

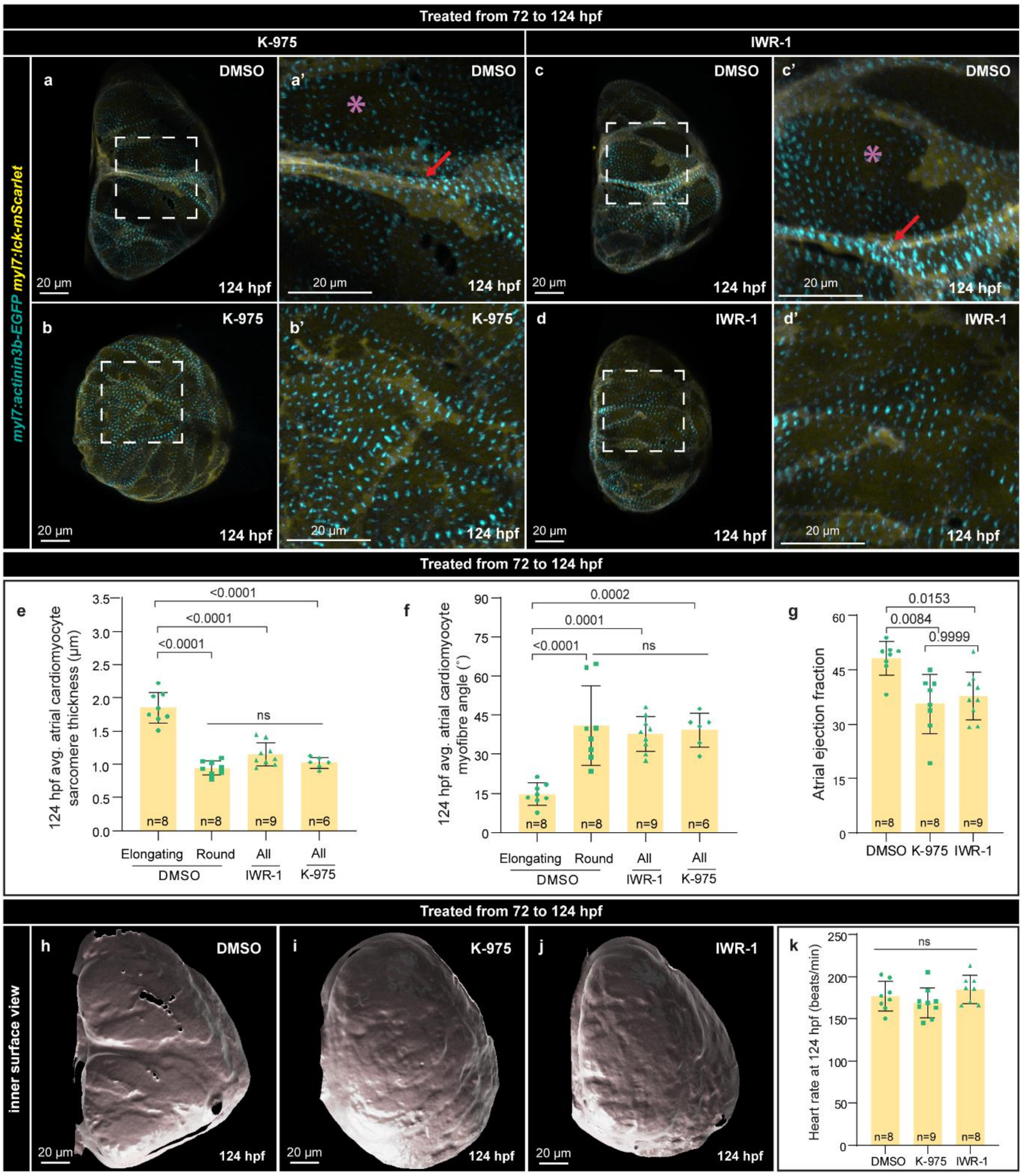
Treated from 72 to 124 hpf
K-975
IWR-1
myl7:actinin3b-EGFP myl7:lck-mScarlet
a DMSO
a' DMSO
c DMSO
c' DMSO
b K-975
b' K-975
d IWR-1
d' IWR-1
20 µm
124 hpf
Treated from 72 to 124 hpf
e
124 hpf avg. atrial cardiomyocyte sarcomere thickness (µm)
<0.0001
<0.0001
<0.0001
ns
n=8 n=8 n=9 n=6
Elongating Round All All
DMSO IWR-1 K-975
f
124 hpf avg. atrial cardiomyocyte myofibre angle (°)
0.0002
0.0001
<0.0001
ns
n=8 n=8 n=9 n=6
Elongating Round All All
DMSO IWR-1 K-975
g
Atrial ejection fraction
0.0153
0.0084
0.9999
n=8 n=8 n=9
DMSO K-975 IWR-1
Treated from 72 to 124 hpf
inner surface view
h DMSO
i K-975
j IWR-1
20 µm
124 hpf
k
Heart rate at 124 hpf (beats/min)
ns
n=8 n=9 n=8
DMSO K-975 IWR-1

Supplementary Figure 11: Loss of atrial cardiomyocyte elongation impairs cardiac function. **a-c,** 3D confocal images of atria from 124 hpf larvae treated from 72 to 124 hpf with DMSO (e, e', g, g'), K-975 (f, f'), or IWR-1 (h, h'); CM membranes shown in yellow (*myl7:lck-mScarlet*) and sarcomere Z-bands in cyan (*myl7:actinin3b-EGFP*). Dashed squares indicate the zoomed in regions; red arrows point to elongating CMs; purple asterisks indicate round CMs. **e,** Atrial CM average sarcomere thickness from 124 hpf larvae treated from 72 to 124 hpf with DMSO, IWR-1, or K-975 (n=8 DMSO; average of 3 round and 3 elongating CMs per heart; n=9 IWR-1, n=6 K-975; average of 6 CMs per heart; each data point represents one heart; ordinary one-way ANOVA with Tukey's multiple comparisons test, DMSO elongating vs round p-value = $0.14*10^{-9}$, DMSO elongating vs IWR-1 p-value = $0.17*10^{-7}$, DMSO elongating vs K-975 p-value = $0.49*10^{-8}$, DMSO round vs IWR-1 p-value = 0.0509, DMSO round vs K-975 p-value = 0.3909, IWR-1 vs K-975 p-value = 0.2941). **f,** Average atrial CM myofibril orientation from 124 hpf larvae treated from 72 to 124 hpf with DMSO, IWR-1, or K-975 (n=8 DMSO; average of 3 round and 3 elongating CMs per heart; n=9 IWR-1, n=6 K-975; average of 6 cells per heart; each data point represents one heart; ordinary one-way ANOVA with Tukey's multiple comparisons test, DMSO elongating vs round p-value = $0.29*10^{-4}$, DMSO round vs IWR-1 p-value = 0.9007, DMSO round vs K-975 p-value = 0.988, IWR-1 vs K-975 p-value = 0.991). **g,** Atrial ejection fraction in 124 hpf larvae treated with DMSO, K-975, or IWR-1 (n=8 DMSO and K-975, n=9 IWR-1, each data point represents one heart; Kruskal-Wallis test with Dunn's multiple comparisons test). **h-j,** Cross-sections through 3D reconstructed atrium from 124 hpf larvae treated from 72 to 124 hpf with DMSO, K-975, or IWR-1. **k,** Heart rate in 124 hpf larvae treated from 72 to 124 hpf with DMSO, K-975, or IWR-1 (n=8 DMSO, n=9 K-975, n=8 IWR-1; each data point represents one heart; ordinary one-way ANOVA with Tukey's multiple comparisons test, DMSO vs K-975 p-value = 0.6385, DMSO vs IWR-1 p-value = 0.628, K-976 vs IWR-1 p-value = 0.1715). Error bars are mean ± SD.

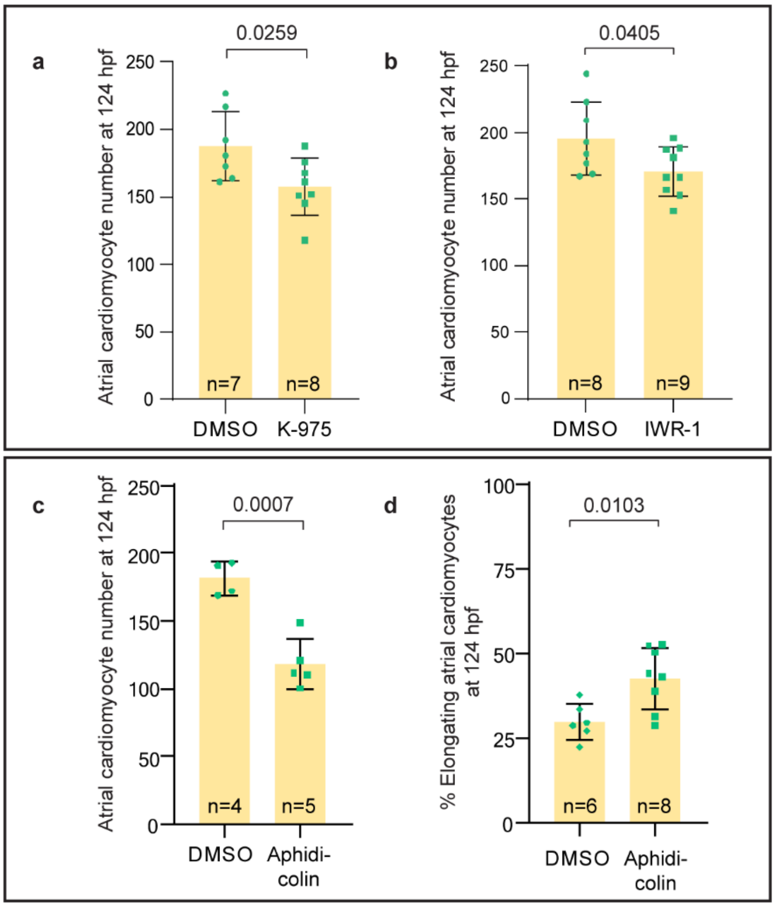

Supplementary Figure 12: Hippo pathway inhibition reduces atrial cardiomyocyte numbers. a, b, Atrial CM numbers in 124 hpf larvae treated from 72 to 124 hpf with DMSO, K-975, or IWR-1 (n=7 DMSO, n=8 K-975, n=8 DMSO, n=9 IWR-1; each data point represents one heart; two-tailed unpaired Student's t-test). **c,** Atrial CM numbers in 124 hpf larvae treated from 72 to 124 hpf with DMSO or Aphidicolin (n=4 DMSO, n=5 Aphidicolin; each data point represents one heart; two-tailed unpaired Student's t-test). **d,** Percentage of elongating atrial CMs in 124 hpf larvae treated from 72 to 124 hpf with DMSO or Aphidicolin (n=6 DMSO, n=8 Aphidicolin, each data point represents one heart; two-tailed unpaired Student's t-test). Error bars are mean ± SD.

Condition	Atrial CM protrusion formation	Atrial CM elongation	Atrial CM orientation
IRSp53^{DN} CM overexpression	reduced	reduced	affected
NSC23766 (Rac1 inhibition)	not reduced	reduced	not affected
MYL9^{DN} CM overexpression	not reduced	reduced	not analyzed
MYL9^{CA} CM overexpression	not analyzed	increased	not analyzed
K-975 (Yap1 inhibition)	not reduced	reduced	affected
IWR-1 (Tankyrase inhibition)	not reduced	reduced	affected

Supplementary Table 1: Relationship between atrial CM protrusions and their elongation.
