# Supplementary material for: Distinct mechanisms regulate ventricular and atrial chamber wall formation
Source: Nat Commun. 2024 Sep 17;15:8159. doi: 10.1038/s41467-024-52340-3 (PMC11408654; doi:10.1038/s41467-024-52340-3)
Supplement: Supplementary file 3 — Description of Additional Supplementary Files [file 41467_2024_52340_MOESM3_ESM.pdf]

Description of Additional Supplementary Files:

Supplementary Movie 1: Complete 3D reconstruction of the zebrafish atrium at 124 hpf. CM membranes shown in cyan (myl7:EGFP-Hsa.HRAS) and CM nuclei in yellow (myl7:H2B-mScarlet); grey spheres indicate the nuclei of all the atrial CMs; cross-section through the atrium shows an example of an elongating atrial CM segmented in 3D with a magenta surface.

Supplementary Movie 2: Atrial muscle structures at 14 dpf. 360º view of the heart shown in Fig. 3 and its 3D segmentations. CM membranes shown in white and cyan (myl7:EGFP-Hsa.HRAS) and CM nuclei in yellow (myl7:H2B-mScarlet); cyan marks the inner layer atrial CMs.
