# Supplementary material for: Distinct mechanisms regulate ventricular and atrial chamber wall formation
Source: Nat Commun. 2024 Sep 17;15:8159. doi: 10.1038/s41467-024-52340-3 (PMC11408654; doi:10.1038/s41467-024-52340-3)
Supplement: Supplementary file 6 — Reporting Summary [file 41467_2024_52340_MOESM6_ESM.pdf]

nature portfolio

Corresponding author(s): Didier Y. R. Stainier

Last updated by author(s): Jul 14, 2024

Reporting Summary

Nature Portfolio wishes to improve the reproducibility of the work that we publish. This form provides structure for consistency and transparency in reporting. For further information on Nature Portfolio policies, see our Editorial Policies and the Editorial Policy Checklist.

Statistics

For all statistical analyses, confirm that the following items are present in the figure legend, table legend, main text, or Methods section.

n/a	Confirmed	
☐	☒	The exact sample size (*n*) for each experimental group/condition, given as a discrete number and unit of measurement
☐	☒	A statement on whether measurements were taken from distinct samples or whether the same sample was measured repeatedly
☐	☒	The statistical test(s) used AND whether they are one- or two-sided<br>*Only common tests should be described solely by name; describe more complex techniques in the Methods section.*
☐	☒	A description of all covariates tested
☐	☒	A description of any assumptions or corrections, such as tests of normality and adjustment for multiple comparisons
☐	☒	A full description of the statistical parameters including central tendency (e.g. means) or other basic estimates (e.g. regression coefficient) AND variation (e.g. standard deviation) or associated estimates of uncertainty (e.g. confidence intervals)
☐	☒	For null hypothesis testing, the test statistic (e.g. *F*, *t*, *r*) with confidence intervals, effect sizes, degrees of freedom and *P* value noted<br>*Give P values as exact values whenever suitable.*
☒	☐	For Bayesian analysis, information on the choice of priors and Markov chain Monte Carlo settings
☒	☐	For hierarchical and complex designs, identification of the appropriate level for tests and full reporting of outcomes
☒	☐	Estimates of effect sizes (e.g. Cohen's *d*, Pearson's *r*), indicating how they were calculated

Our web collection on statistics for biologists contains articles on many of the points above.

Software and code

Policy information about availability of computer code

Data collection	Zen 2.3 SP1 FP3 black, Zen 2011 SP3 black, Zen 2.6 Blue were used for imaging.
Data analysis	Zen 2.3 SP1 FP3 black, Imaris 10.0.0, Imarisfile Converter 10.0.0 and ImageJ were used for image analysis; GraphPad Prism 9 for statistical tests; R for polar plots.

For manuscripts utilizing custom algorithms or software that are central to the research but not yet described in published literature, software must be made available to editors and reviewers. We strongly encourage code deposition in a community repository (e.g. GitHub). See the Nature Portfolio guidelines for submitting code & software for further information.

Data

Policy information about availability of data

All manuscripts must include a data availability statement. This statement should provide the following information, where applicable:

- Accession codes, unique identifiers, or web links for publicly available datasets
- A description of any restrictions on data availability
- For clinical datasets or third party data, please ensure that the statement adheres to our policy

The bulk RNA-sequencing dataset reported in this paper was deposited in the Gene Expression Omnibus (GEO) database (accession: GSE249149 [https://www.ncbi.nlm.nih.gov/geo/query/acc.cgi?acc=GSE249149]).
Source data are provided with the paper.

Research involving human participants, their data, or biological material

Policy information about studies with human participants or human data. See also policy information about sex, gender (identity/presentation), and sexual orientation and race, ethnicity and racism.

Reporting on sex and gender	*Use the terms sex (biological attribute) and gender (shaped by social and cultural circumstances) carefully in order to avoid confusing both terms. Indicate if findings apply to only one sex or gender; describe whether sex and gender were considered in study design; whether sex and/or gender was determined based on self-reporting or assigned and methods used. Provide in the source data disaggregated sex and gender data, where this information has been collected, and if consent has been obtained for sharing of individual-level data; provide overall numbers in this Reporting Summary. Please state if this information has not been collected. Report sex- and gender-based analyses where performed, justify reasons for lack of sex- and gender-based analysis.*
Reporting on race, ethnicity, or other socially relevant groupings	*Please specify the socially constructed or socially relevant categorization variable(s) used in your manuscript and explain why they were used. Please note that such variables should not be used as proxies for other socially constructed/relevant variables (for example, race or ethnicity should not be used as a proxy for socioeconomic status). Provide clear definitions of the relevant terms used, how they were provided (by the participants/respondents, the researchers, or third parties), and the method(s) used to classify people into the different categories (e.g. self-report, census or administrative data, social media data, etc.) Please provide details about how you controlled for confounding variables in your analyses.*
Population characteristics	*Describe the covariate-relevant population characteristics of the human research participants (e.g. age, genotypic information, past and current diagnosis and treatment categories). If you filled out the behavioural & social sciences study design questions and have nothing to add here, write "See above."*
Recruitment	*Describe how participants were recruited. Outline any potential self-selection bias or other biases that may be present and how these are likely to impact results.*
Ethics oversight	*Identify the organization(s) that approved the study protocol.*

Note that full information on the approval of the study protocol must also be provided in the manuscript.

Field-specific reporting

Please select the one below that is the best fit for your research. If you are not sure, read the appropriate sections before making your selection.

☒ Life sciences ☐ Behavioural & social sciences ☐ Ecological, evolutionary & environmental sciences

For a reference copy of the document with all sections, see nature.com/documents/nr-reporting-summary-flat.pdf

Life sciences study design

All studies must disclose on these points even when the disclosure is negative.

Sample size	In compliance with the principle of the 3Rs we sought to reduce the number of animals as far as possible. We determined sample size through a Power test on data from animals younger than 120 hours post fertilization (hpf) (before the legally protected stages are reached). Using these data we determined a maximum of 13 animals 120 hpf or older to be used per condition which was accepted under the animal proposal.
Data exclusions	For all longitudinal imaging, zebrafish that did not recover well after imaging were excluded from analysis and further imaging. silent atrium mutants (Supplementary Fig. 4m) which had absent blood flow due to a collapsed ventricle were excluded from imaging and analysis.
Replication	All experiments were performed on at least three independent batches of animals.
Randomization	For all experiments (except those involving mutants), the embryos were sorted for fluorescence and healthy morphology, before being randomly assigned into experimental groups.
Blinding	Blinding procedures were performed whenever possible, except for drug treatments as the experiment and data analysis were performed by the same investigator.

Reporting for specific materials, systems and methods

We require information from authors about some types of materials, experimental systems and methods used in many studies. Here, indicate whether each material, system or method listed is relevant to your study. If you are not sure if a list item applies to your research, read the appropriate section before selecting a response.

Materials & experimental systems

n/a	Involved in the study
	x Antibodies
x	Eukaryotic cell lines
x	Palaeontology and archaeology
	x Animals and other organisms
x	Clinical data
x	Dual use research of concern
x	Plants

Methods

n/a	Involved in the study
x	ChIP-seq
	x Flow cytometry
x	MRI-based neuroimaging

Antibodies

Antibodies used	Anti-Yap1 polyclonal antibody (generated by Prof. Virginie Lecaudey, 1:200), anti-eGFP (monoclonal chicken, ab13970, 1:200), Alexa Fluor 488 and Alexa Fluor 647 (produced in goat, Thermo Fisher Scientific, 1:500).
Validation	Antibodies were validated by suppliers or in other published peer-reviewed studies.<br><br>Anit-Yap1<br>Bornhorst, D. et al. Biomechanical signaling within the developing zebrafish heart attunes endocardial growth to myocardial chamber dimensions. Nat Commun 10, 4113 (2019).<br>Peralta, M. et al. Intraflagellar Transport Complex B Proteins Regulate the Hippo Effector Yap1 during Cardiogenesis. Cell Reports 32, 107932 (2020)<br><br>anti-eGFP<br>Gunawan, F. et al. Focal adhesions are essential to drive zebrafish heart valve morphogenesis. J Cell Biol 218, 1039-1054, doi:10.1083/jcb.201807175 (2019).

Animals and other research organisms

Policy information about studies involving animals; ARRIVE guidelines recommended for reporting animal research, and Sex and Gender in Research

Laboratory animals	Zebrafish (Danio rerio), strain: Tüb/AB, adult fish (both male and females) were used in the study. Experiments were performed on animals up to 14 days post fertilization, sex is not specified.
Wild animals	No wild animals were used in this study.
Reporting on sex	Sex is not specified at the time of experiments, and thus was not taken into consideration.
Field-collected samples	No field-collected samples were used in this study.
Ethics oversight	All procedures performed on animals conform to the guidelines from Directive 2010/63/EU of the European Parliament on the protection of animals used for scientific purposes and were approved by the Animal Protection Committee (Tierschutzkommission) of the Regierungspräsidium Darmstadt (reference: B2/2057). Statistical experiments performed before 120 hpf to determine a maximum sample size to be used on larvae past 120 hpf. Sample size was calculated to minimize the number of animals used in compliance to the principle of the 3Rs and the European Directive 2010/63/EU.

Note that full information on the approval of the study protocol must also be provided in the manuscript.

Plants

Seed stocks	*Report on the source of all seed stocks or other plant material used. If applicable, state the seed stock centre and catalogue number. If plant specimens were collected from the field, describe the collection location, date and sampling procedures.*
Novel plant genotypes	*Describe the methods by which all novel plant genotypes were produced. This includes those generated by transgenic approaches, gene editing, chemical/radiation-based mutagenesis and hybridization. For transgenic lines, describe the transformation method, the number of independent lines analyzed and the generation upon which experiments were performed. For gene-edited lines, describe the editor used, the endogenous sequence targeted for editing, the targeting guide RNA sequence (if applicable) and how the editor was applied.*
Authentication	*Describe any authentication procedures for each seed stock used or novel genotype generated. Describe any experiments used to assess the effect of a mutation and, where applicable, how potential secondary effects (e.g. second site T-DNA insertions, mosiacism, off-target gene editing) were examined.*

Flow Cytometry

Plots

Confirm that:

- [x] The axis labels state the marker and fluorochrome used (e.g. CD4-FITC).
- [x] The axis scales are clearly visible. Include numbers along axes only for bottom left plot of group (a 'group' is an analysis of identical markers).
- [x] All plots are contour plots with outliers or pseudocolor plots.
- [x] A numerical value for number of cells or percentage (with statistics) is provided.

Methodology

Sample preparation

Approximately 150 hearts at 48 and 72 hpf were extracted through manual dissection and dissociated using the Pierce Cardiomyocyte Isolation Kit (Thermo Fisher Scientific, Catalog# 88281) as previously described97. In brief, hearts were dissected in Dulbecco's modified Eagle's medium (DMEM)+GlutaMAX (Thermo Fisher Scientific, Catalog# 10566016) supplemented with 10% FBS and kept on ice throughout the dissociation protocol; the hearts were centrifuged at 4ºC for 5 minutes at 2300g; the supernatant was removed and the hearts were washed with 1 ml Hank's Balanced Salt Solution (HBSS); the tissue was dissociated into single cells by incubating it with 100 µl Enzyme 1 and 5 µl Enzyme 2 from the Pierce Cardiomyocyte Isolation Kit at 30ºC on a shaker set at 300 rpm; 1 ml of DMEM with FBS was added to stop the digestion and removed after centrifugation at 4ºC for 3 min at 800 g; fresh DMEM with FBS was added to resuspend the cells and pass them through a 40 µl-filtered fluorescence-activated cell sorting (FACS) sample tube. 2 µl of DRAQ7™ Dye (Catalog# D15106) was added and incubated in the dark for 10 min at RT. The cell suspension was filtered through a 35 µm nylon Falcon® 5 mL Round Bottom Polystyrene 12x75 mm Test Tube (Product# 352235). Cells were sorted using a BD FACSAria™ III (BD Biosciences) or an Invitrogen Bigfoot Spectral Cell Sorter (ThermoFisher Scientific) equipped with a 100 µM nozzle and with 20 psi pressure on the instruments. Live and non-AVC and–SAN CMs were gated by exclusion of DRAQ7™ Dye using 633 nm excitation paired with 730/45 nm band pass filter and eGFP fluorescence using 488 nm excitation paired with 530/30 nm band pass filter, respectively. To sort atrial CMs (myh6:mTagBFP2+), tagBFP fluorescence was measured with 405 nm excitation paired with 455/14 nm band pass filter or 450/50 nm band pass filter; to sort ventricular CMs (myh7:mCherry-NTR +), mCherry fluorescence was measured with 561 nm excitation paired with 610/20 nm band pass filter. Sorted cells were resuspended in 500 µl Trizol for subsequent RNA extraction.

Instrument

BD FACSAria™ III and BigFoot

Software

BD FACSAria™ III and BigFoot

Cell population abundance

Embryonic zebrafish atrial CMs are low in number and sensitive to dissociation, so in order to get enough cells for RNA-sequencing, a total of 5 sorting sessions were completed to obtain 1000-2000 cells per replicate, 3 replicates per condition. The first two replicates consist of cells sorted using the BD FACSAria™ III and BigFoot and the third replicate consists of cells sorted using the BigFoot only.

Purity was approximately 95%

Gating strategy

For gating (Supplementary Fig. 5e-h''), following exclusion of debris, cell population was selected (Cells), from which single cells were gated with FSC-A vs FSC-H parameter (Single cells). Within this single cell population, live cells were selected by exclusion of DRAQ7™ Dye+ population and eGFP+ AVC and SAN CMs. High intensity mTagBFP2 cells with low or no mCherry signal was used to gate Atrial CMs. Ventricular CMs were gated for only mCherry positive cells as low mCherry signal was present in the atrium, and high mCherry in the ventricle (Supplementary Fig. 5a-d).

- [x] Tick this box to confirm that a figure exemplifying the gating strategy is provided in the Supplementary Information.
